# To fill or not to fill: a qualitative cross-country study on dentists’ decisions in managing non-cavitated proximal caries lesions

**DOI:** 10.1186/s13012-018-0744-7

**Published:** 2018-04-06

**Authors:** F. Schwendicke, L. A. Foster Page, L. A. Smith, M. Fontana, W. M. Thomson, S. R. Baker

**Affiliations:** 1 Department for Operative and Preventive Dentistry, Charité – Universitätsmedizin Berlin, Freie Universität Berlin, Humboldt-Universität zu Berlin, and Berlin Institute of Health, Aßmannshauser Str. 4-6, 14197 Berlin, Germany; 2 Department of Oral Sciences, Faculty of Dentistry, University of Otago, Dunedin, New Zealand; 3 Department of Cariology, Restorative Sciences and Endodontics, School of Dentistry, University of Michigan, Ann Arbor, USA; 4 Unit of Oral Health, Dentistry and Society, School of Clinical Dentistry, University of Sheffield, Sheffield, UK

**Keywords:** Attitudes, Dental, Decision-making, Enamel caries, Evidence-based practice, Qualitative studies, Theoretical Domains Framework

## Abstract

**Background:**

This study aimed to identify barriers and enablers for dentists managing non-cavitated proximal caries lesions using non- or micro-invasive (NI/MI) approaches rather than invasive and restorative methods in New Zealand, Germany and the USA.

**Methods:**

Semi-structured interviews were conducted, focusing on non-cavitated proximal caries lesions (radiographically confined to enamel or the outer dentine). Twelve dentists from New Zealand, 12 from Germany and 20 from the state of Michigan (USA) were interviewed. Convenience and snowball sampling were used for participant recruitment. A diverse sample of dentists was recruited. Interviews were conducted by telephone, using an interview schedule based on the Theoretical Domains Framework (TDF).

**Results:**

The following barriers to managing lesions non- or micro-invasively were identified: patients’ lacking adherence to oral hygiene instructions or high-caries risk, financial pressures and a lack of reimbursement for NI/MI, unsupportive colleagues and practice leaders, not undertaking professional development and basing treatment on what had been learned during training, and a sense of anticipated regret (anxiety about not restoring a proximal lesion in its early stages before it progressed). The following enablers were identified: the professional belief that remineralisation can occur in early non-cavitated proximal lesions and that these lesions can be arrested, the understanding that placing restorations weakens the tooth and inflicts a cycle of re-restoration, having up-to-date information and supportive colleagues and work environments, working as part of a team of competent and skilled dental practitioners who perform NI/MI (such as cleaning or scaling), having the necessary resources, undertaking ongoing professional development and continued education, maintaining membership of professional groups and a sense of professional and personal satisfaction from working in the patient’s best interest. Financial aspects were more commonly mentioned by the German and American participants, while continuing education was more of a focus for the New Zealand participants.

**Conclusions:**

Decisions on managing non-cavitated proximal lesions were influenced by numerous factors, some of which could be targeted by interventions for implementing evidence-based management strategies in practice.

**Electronic supplementary material:**

The online version of this article (10.1186/s13012-018-0744-7) contains supplementary material, which is available to authorized users.

## Background

Dental caries is highly prevalent and costly to treat [1, 2]. Traditionally, caries has been treated by the surgical removal of all carious tissue from the tooth and the placement of a restoration in the resulting cavity. Contemporary management aims to control disease activity non-invasively (NI), without breaching the surface of the tooth (by restricting cariogenic sugars intake, avoiding biofilm maturation or remineralising) [3–5]. Alternatively, micro-invasive (MI) therapies can be applied, with only a few micrometres of tooth tissue being removed, usually during a conditioning step (using acid). Sealing caries lesions or infiltrating them using resins are such micro-invasive therapies. They impede bacterial acid diffusion into the tooth tissues, arresting the lesion [6].

NI/MI control caries lesions without placing restorations [4, 7] and are currently recommended for managing early, non-cavitated caries lesions [6, 8–10]. However, dentists have not unequivocally adopted NI/MI [11]; instead, most continue to intervene invasively even for non-cavitated lesions [12–14]. To understand how factors such as knowledge, skills, attitudes or behaviours shape dentists’ decision-making, qualitative studies are needed; those could then inform interventions to promote evidence-based management for non-cavitated caries lesions [15]. Such studies should use theoretical frameworks [16].

Here, we used the Theoretical Domains Framework (TDF), which has been used previously to understand the motivations, cognitions and behaviours of health professionals when implementing evidence-based practice [17–19]. Few dental studies have used it [17, 20, 21]. Since interventions for implementing behaviour change must be setting-specific, we aimed to identify barriers and enablers to dentists non- or micro-invasively managing proximal caries lesions in New Zealand (NZ), the USA, and Germany. These countries were chosen for pragmatic reasons but were well suited to contrast barriers and enablers in three different dental healthcare systems.

## Methods

Semi-structured interviews covered seven areas: (1) the dentist’s background (e.g. years of experience), (2) current clinical practice and decision-making for NI/MI/restoration, (3) knowledge and skills (e.g. awareness of guidelines), (4) professional role and identity (e.g. whose responsibility is NI/MI), (5) goals (e.g. changes to routine management in the future), (6) beliefs about consequences (e.g. benefits/disadvantages of NI/MI), (7) environmental context (e.g. factors that influence NI/MI), and (8) social influences (e.g. patient choice) (Additional file 1 for interview schedule). Our reporting follows the COREQ checklist [22]. Ethical approval was obtained (see “Ethics approval and consent to participate” section).

### Research team and reflexivity

An experienced psychologist and qualitative researcher (SB) led the design of the interview schedule and data analysis. The clinical and dental research expertise was led by three researchers, one from each country (FS, LFP, MF). All researchers (a health psychologist, two cariologists, a dental public health specialist and a sociologist) acted as an expert panel to determine how to apply the TDF to oral healthcare [17]. Interviews were conducted by three dental researchers with a previous experience or trained via pilot interviews (see below). Those were conducted in the second half of 2016, in order to assess interview schedule suitability and provide feedback to interviewers. A dental researcher with a previous experience in qualitative research (LS) coded the interviews; 10% were additionally and independently coded by SB. The interviewees did not have any relationship with the interviewers; all were informed about the study’s purpose before consenting.

### Theoretical framework and methodology

Providing the underlying structure for data collection, analysis and interpretation, the TDF comprises 14 theoretical domains, each having a number of different constructs [23]. Ten of the 14 TDF domains were utilised in this study (Table 1) because these were felt by the expert panel to be the most relevant.

**Table 1:** Domains of the Theoretical Domains Framework (TDF)

Domain	Construct	Definition
Knowledge	Knowledge	Knowledge of a condition or scientific rationale.
	Procedural knowledge	Knowing how to do something.
	Knowledge of task environment	Knowledge of the social and material context in which a task is undertaken.
Skills	Skills	An ability or proficiency acquired through practice.
	Skills development	The gradual acquisition or advancement through progressive stages of an ability or proficiency acquired through training and practice.
	Competence	One’s repertoire of skills and ability especially as it is applied to a task or set of tasks.
	Ability	Competence or capacity to perform a physical or mental act. Ability may be either learned or unlearned.
	Interpersonal skills	An aptitude enabling a person to carry on effective relationships with others, such as ability to cooperate, to assume appropriate relationships with others or to exhibit adequate flexibility.
	Practice	Repetition of an act, behaviour or series of activities, often to improve performance or acquire a skill.
Social influences	Social pressure	The exertion of influence on a person or person or group by another person or group.
	Social norms	Socially determined consensual standards that indicate what behaviours are considered typical in a given context and what behaviours are considered proper in the context.
	Group conformity	The act of consciously maintaining a certain degree of similarity to those in your general social circle.
	Social comparisons	The process by which people evaluate their attitudes, abilities, or performance relative to others.
	Group norms	Any behaviour, belief, attitude or emotion reaction held to be correct by any given group in society.
	Social support	The apperception or provision of assistance or comfort to others, typically in order to help them to cope with a variety of biological, psychological or social stressors. Support may arise from interpersonal relationships in an individual’s social network, involving friends, neighbours, religious institutions, colleagues, caregivers or support groups.
	Power	The capacity to influence others, even when they try to resist this influence.
	Intergroup conflict	Disagreement or confrontation between two or more groups and their members. This may involve physical violence, interpersonal discord, or psychological tension.
	Alienation	Estrangement from one’s social group; a deep seated sense of dissatisfaction with one’s personal experiences that can be a source of lack of trust in one’s social or physical environment or in oneself; the feeling of separation between one’s thoughts and feelings.
	Group identity	The set of behaviour or personal characteristics by which an individual is recognisable (and portrays) as a member of a group.
	Modelling	In developmental psychology, the process by which one or more individuals or other entities serve as examples (models) that a child will copy.
Social/ professional role and identity	Professional identity	The characteristics by which an individual is recognised relating to, or connected with, or benefitting, a particular profession.
	Professional role	The behaviour considered appropriate for a particular kind of work or social position.
	Social identity	The set of behaviours or personal characteristics by which an individual is recognisable [and portrays] as a member of a social group, relating to, or connected with or benefitting a particular profession
	Identity	An individual’s sense of self defined by (a) a set of physical and psychological characteristics that is not wholly shared with any other person and (b) a range of social and interpersonal affiliations (e.g. social roles).
	Professional boundaries	The bounds or limits relating to, or connected with, a particular profession or calling.
	Professional confidence	An individual’s beliefs in his or her repertoire of skills and ability as it is applied to tasks or set of tasks.
	Group identity	The set of behaviours or personal characteristics by which an individual is recognisable [and portrays] as a member of a group.
	Leadership	The process involved in leading others, including organising directing, coordinating and motivating their efforts toward achievement of certain group or organisational goals.
Beliefs about consequences	Beliefs	The thing one believed in, the proposition or set of propositions held true.
	Outcome expectancies	Cognitive, emotional, behavioural and affective outcomes that are assumed to be associated with future or intended behaviours. These assumed outcomes can either promote or inhibit future behaviour.
	Characteristics of outcome expectancies	Characteristics of the cognitive, emotional and behavioural outcomes that individuals believe are associated with future or intended behaviours and that are either believed to promote or inhibit these behaviours. These include whether they are sanctions/rewards, proximal/distal, valued/not valued, probable/improbable, salient/not salient, perceived risks or threats.
Reinforcement	Anticipated regret	A sense of the negative consequences of a decision that influences the choice made; for example, an individual may decide not to make an investment because of the feelings associated with an imagined loss.
	Consequence	An outcome of behaviour in a given situation.
	Rewards	Return or recompense, made to or received by a person contingent on some purpose.
	Incentives	An external stimulus, such as a condition or object that enhances or serves as a motive for behaviour.
	Punishment	The process in which a relationship between a response and some stimulus or circumstance results in the response becoming less probable; a painful, unwanted or undesired event or circumstance imposed on a wrong doer.
	Consequents	An outcome of behaviour in a given situation.
	Reinforcement	A process in which the frequency of a response is increased by a dependent relationship or contingency with a stimulus.
	Contingencies	A conditional probabilistic relation between two events. Contingencies may be arranged via dependencies or they emerge by accident.
	Sanctions	A punishment or other coercive measure, usually administered by a recognised authority, that is used to penalise and deter inappropriate or unauthorised actions
Intentions	Stability of intentions	Ability of one’s resolve to remain in spite of disturbing influences.
	Stages of change model	A model that proposes that behaviour change is accomplished through five specific stages:- pre-contemplation, contemplation, preparation, action and maintenance.
	Trans-theoretical model and stages of change	A five-stage theory to explain changes in people’s health behaviour. It suggests that change takes time, that different interventions are effective at different stages, and that there are multiple outcomes occurring across different stages.
Goals	Goals (distal/proximal)	Desired state of affairs of a person or system; these may be closer (proximal) or further away (distal).
	Goal priority	Order of importance or urgency of end states toward which one is striving.
	Goal/target setting	A process that establishes specific time based behaviour targets that are measurable, achievable and realistic.
	Goals (autonomous/controlled)	The end state towards which one is striving: the purpose of an activity or endeavour. It can be observed by observing that a person ceases or changes its behaviour upon attaining this state; proficiency in a task to be achieved within a set period of time.
	Action planning	The action or process of forming a plan regarding a thing to be done or a deed.
	Implementation intention	The plan that creates in advance of when, where and how one will enact a behaviour.
Environmental context and resources	Environmental stressors	External factors in the environment that cause stress.
	Resources material resources	Commodities and human resources used in enacting behaviour.
	Organisational culture/climate	A distinctive pattern of thought and behaviour shared by members of the same organisation and reflected in their language, values, attitudes, beliefs and customs.
	Salient events/critical incidents	Occurrences that one judges to be distinctive, prominent or otherwise significant.
	Person–environment interaction	Interplay between an individual and their surroundings.
	Barriers and facilitators	In psychological contexts barriers/facilitators are mental, emotional or behavioural limitations/strengths in individuals or groups.
Behaviour regulation	Self-monitoring	A method used in behaviour management in which individuals keep a record of their behaviour, especially in connection with efforts to change or regulate the self; a personality trait reflecting an ability to modify one’s behaviour in response to a situation.
	Breaking habit	To discontinue a behaviour or sequence of behaviours that is automatically activated by relevant situational cues.
	Action planning	The action or forming of a plan regarding a thing to be done or a deed.

### Participant selection

NZ, the USA and Germany were chosen primarily for pragmatic reasons, including research team location and cost minimisation; they also represented three very different dental healthcare systems. In the USA, we sampled from only one state (Michigan) because it was felt that it would not be possible to use a USA-wide sample of dentists. We chose accordingly to sample from a state with many of the public and private healthcare systems available throughout the country. In each country, lists of registered dentists supplied names of general dentists who were then informed about the study by the research assistant or assistant research fellow in that country via email or telephone. Informal approaches and snowballing were also used.

Although there is no specific requirement for the number of participants in qualitative research [24], we wanted to gather a diverse range of general dentists (by gender, age, time since graduation, type of practice, rural/urban) and collect sufficiently broad data to understand barriers and enablers within each country. We continued recruiting in each country until no new data or no new patterns or themes emerged [25]. Our final sample comprised 44 participants (12 in NZ, 12 in Germany, 20 in the USA; Table 2). The characteristics of non-responders were not recorded separately, and reasons for non-response were not sought.

**Table 2:** Summary of participant demographic characteristics

Country	Sex	Mean (range) years of experience*	Location of practice	Type of practice	Case mix (reimbursement scheme)
USA––Michigan (*n* = 20)	5 female 15 male	20 (1–42)	19 urban 1 rural	12 group 5 sole 3 unknown	6 private only 12 mixed 1 insurer 1 not disclosed
New Zealand (*n* = 12)	5 female 7 male	26 (6–47)	9 urban 3 rural	9 group 3 sole	All fee for service
Germany (n = 12)	7 female 5 male	18 (1–41)	8 urban 4 rural	9 group 3 sole	Predominantly statutory health insurance
Total (*n* = 44)	17 female 27 male	22 (1–47)*	36 urban 8 rural	30 group 11 sole 3 unknown	

*Excluding two participants whose experience had not been recorded

### Data collection

We combined some TDF domains to reduce the time burden on participants (Additional file 1). The interview schedule contained a mixture of close- and open-ended questions. Conducted by telephone and audio-recorded, interviews lasted 20–60 min. In NZ and Michigan, participants received a $50 voucher as a token of appreciation. These interviews were transcribed by a professional transcription service, while a native German speaker residing in NZ transcribed the German interviews and translated them into English. The accuracy of the German-to-English translation was double-checked by another German native speaker against the original audio recording.

### Data analysis

The data were analysed systematically using a deductive approach [26], with the TDF as the framework. The researcher (LS) first read the participant’s response and considered it in relation to each TDF domain and its constructs. It would then be attributed to one domain. A sample of the coding across domains was independently coded by a second researcher (SB) to check for accuracy and consistency. If there was disagreement, it was discussed. If agreement could not be reached, the response was coded into both domains. A response not fitting any domain was noted separately (a rare occurrence). The main coder (LS) then highlighted both exemplary quotes for each construct for each country, and any inter-country discrepancies in responses. A simple count of the excerpts grouped under domain constructs and the number of participants per country mentioning those was also undertaken, to provide an overall picture of a domain’s pervasiveness without implying validity of the domain or constructs within it. When > 10 participants mentioned a particular construct, it was further broken down into themes.

## Results

Exemplars of comments within each domain are presented. A more detailed description of responses within constructs and domains can be seen by country in Additional file 1.

### Knowledge

Most dentists made comments regarding knowledge, with slightly more relating to procedural than construct knowledge. The former all centred on how the participants treated their patients using NI/MI, with most focusing on NI.

*Usually I would look at it first. Afterwards I would use a probe to check whether or not the enamel is still intact. If in doubt I would take a bitewing x-ray. If the surface is still intact then I opt for non-invasive measures, regular recalls prophylaxis and fluoride treatment. (G2)*

Comments grouped under knowledge of the task environment reflected knowledge of their patients’ dental/oral health history and how “compliant” they were perceived to be:

*At the enamel dentine junction, I have to think more about what this patient is like… how acidic is their mouth, how compliant are they going to be with the products. If they’re not interested in the products at all and they have terrible oral hygiene, then … we would need to do other things… non-invasive measures are not going to work. (NZ3)*

Knowledge construct comments generally linked with knowledge of the scientific literature, etc.

*If those new researches/findings remain out there for a few years, then yes. If they are only short-term studies, that have only been around for a year, then I would be very reluctant [to try them]. However, attending advanced courses and reading specialist journals helps to keep up to date. (G9)*

A number also mentioned the importance of continuing education and reading dental journals. By contrast, a number said that they based their treatment on what they had learned at dental school. This was mainly the case with the Michigan and German dentists, who also reported that they did not undertake any ongoing education or professional development.

*I’d like to learn more about other modalities of treatment as well, more than I know. I don’t feel like I’ve had, because that’s not something that was taught when I was at dental school, so … I feel like I am a little short on knowledge or up-to-date with what the latest scientific recommendations are. (USA6)*

*Interviewer: Are your recommendations supported by studies?*

*I believe so because this is what I was told at university. (G2)*

### Skills

Few comments could be grouped under “skills”. Some listed fluoride application, but participants also commented on dietary advice, flossing and general oral hygiene. Comments falling under skills related primarily to experience, with many reporting that their clinical knowledge and professional judgement on using NI/MI increased with time in practice.

*I mean you don’t come out of Dental School having done lots…of things. You have done a few of many things, and so you, the real learning starts, I mean certainly from personal experience in that first year out, where you become exposed to techniques you know, modern and preventive techniques that you might not have even heard of. I came out of Dental School and really it was brush teeth, floss your teeth and we will paint fluoride stuff on, but we have come a long way since then. (NZ8)*

*A lot of it is your experience you’ve built up over a period of time and sometimes I’m right and sometimes I could be wrong. So but I follow my best judgement. But it’s not a written list in essence. It is the 36 years of experience I have in dealing with the management of caries. (USA18)*

### Social influences

All commented under this domain. Most comments fell under the social pressure construct, with patient “compliance” and oral hygiene as pressure factors influencing their decision to use NI/MI. There were 72 mentions of issues related to patient “compliance” (adherence to the dentist’s instructions).

*Well that is the compliance. If he (patient) is willing to come in for regular recalls, do his homework, meaning proper tooth brushing at home and interdental hygiene. Also to brush his tongue, mucosa of the cheeks and to now and then use antibacterial mouthwash [and] if that isn’t the case and the patient does not think it is important to brush his/her teeth, then it can certainly be the case that we will directly do a filling. (G2)*

*The only factor that would influence us to not offer treatment would be if somebody was really not interested and they didn’t show any signs of interest and we consider that it is going to be a waste of time. But you do get some patients or some children that they are not going to do what you ask them and I think that becomes a problem. (NZ10)*

Next was financial pressure, mostly from the German (23 mentions), followed by Michigan (18 mentions) and then NZ participants (2 mentions). All Germans mentioned the statutory German insurance and its lack of financial reimbursement for NI/MI. Many of the Michigan dentists mentioned the US public health insurance not covering NI/MI, with this causing them financial stress.

*Well you know, unfortunately the financial advantage is kind of a negative because I'm financially advantaged to go in and do invasive care, I’m not saying that that’s a good thing. (USA8)*

Wider professional organisations and participants comparing their practice to that of other dentists were also commonly mentioned (social comparisons). More German (11 from 12) and Michigan participants (17 from 20) compared their practice to other dentists than NZ participants (six from 12).

*Well if I do a filling then I will get paid. If I don’t do it, and tell the patient, we will observe (the dental situation), then I don’t get any money. At least a lot less and yes, that is why one or other colleagues tend to… do a filling, even though one may have easily been able to just observe it. (G2)*

*Well I think I’m seeing, you know in slow economic times, I think unfortunately we see a lot of these restoration, these lesions restored when they otherwise could’ve been treated by other means, unfortunately that happens so that’s not really a benefit to the patient but more of a benefit to the provider. (USA12)*

### Social professional role and identity

Most comments were grouped under the professional role construct, followed by professional identity, while fewer fell under social identity and none under identity. Professional identity comments centred on membership of professional bodies, and being knowledgeable about and adhering to evidence or guidelines. Group identity statements pertained to relationships with dental peers. Most comments categorised under professional boundaries centred on economic factors; that is, not engaging in specific treatments for financial profit.

*There are certain colleagues that go by how much they get paid off it, but would probably also do worse fillings. I am sure it is mixed. I don’t know this for sure. To be honest I don’t really like to talk about how much it costs. (G10)*

Comments grouped under professional role were varied, although most covered “educating” patients about good oral health or hygiene practices. Clearly, many participants viewed it as part of their professional role to raise patient awareness of good oral hygiene. It is interesting to note, however, that a few dentists made comments linking carrying out NI measures with hygienists, prophylaxis assistants or nurses.

### Beliefs about consequences

The largest number of comments was grouped under beliefs, followed by outcome expectancies and characteristics of outcome expectancies. The overwhelming majority of comments related to the disadvantages of treating non-cavitated caries lesions with restorations. Almost all of the NZ and German participants and nearly half of the Michigan participants stated that restorations weakened the tooth structure, starting the cycle of restoration replacements. The following were typical.

*The advantage is the preservation of the tooth structure. We will preserve the tooth without a filling because even the best filling isn't the greatest compared to an untouched enamel layer. (G2)*

*I think the biggest benefit is…my belief is that there is nothing better for the tooth than its own natural tooth structure with the filling in place once it is… we know that it has got a certain life expectancy that eventually that filling is going to break down and a bigger filling has to be placed and then a crown has to be placed and it is starting that process for more damage to the tooth. (USA1)*

Many dentists saw NI/MI as a way of avoiding restoring non-cavitated proximal lesions. A number also reported that these methods could lead to remineralisation or lesion arrest.

*I have a lot of lesions that are in enamel only that I see that my colleagues would just treat immediately and I think that there’s potential for remineralisation with getting people better in their hygiene and their diet and trying to reduce the acid injury to the enamel there, and I think there’s a chance for natural remineralisation if the acid injury is arrested, slowed down anyway by different factors like fluoride and hygiene and dietary regulation. (G9)*

At the same time, however, a large number of comments were grouped under anticipated regret (11 NZ, 9 German, 5 USA), that is, not being able to arrest lesions via NI/MI when still small and seeing them progress subsequently to requiring larger restorations. This was often associated with comments related to patients’ poor oral health attitudes and/or “non-compliance”.

*Sometimes you will see a cavity and it is small and you think, oh I should drill that and then you second guess yourself and say, you shouldn’t. Then two years later they are back again and you think, oh thank goodness, I didn’t drill that and other times there is a massive cavity there and you bitterly regret your decision. (NZ1)*

### Reinforcement

Most comments here were grouped under incentives, followed by rewards and reinforcement. Interestingly, very few (but all from Michigan) were grouped under sanctions, punishment and contingencies. As incentives, the benefits of non-invasive measures—such as remineralisation, not destroying tooth structure—were mentioned. As rewards, professional satisfaction and peace of mind were identified, along with enhanced dentist-patient relationships. Some Michigan dentists made comments that suggested they achieved professional satisfaction from treating patients with NI/MI. A few also listed having more patients as a benefit of using NI/MI, and four mentioned enhanced relationships with patients.

*Because one will end up having more satisfied patients and a better bond with the patients. Patients are also a multiplication factor, [as] they recommend [me] to others and then I will have more satisfied patients in the practice… work is a lot more fun that way. (G5)*

*I mean in terms of benefits I guess it’s fulfilling…as a career can be in that I honestly feel as though I’m helping people be truly healthy. I think and the staff feels that, so that would be the main benefit there in terms of being fulfilled in regards to our careers. (USA7)*

### Intentions

Only a few comments were associated with intentions (all under stability of intentions). These generally centred on the desire to use NI/MI whenever possible. Most stated that they would use NI in the outer half of the enamel, but fewer would if a caries lesion had reached the dentin-enamel junction. Most would alter their management strategy if patients had poor oral hygiene or if they felt patients would not follow their hygiene instructions.

*Yes, definitely a priority for us in our practice. I do feel that, the less you do to the tooth the better chance that you have surviving longer, because as soon as you drill the tooth, really every couple of years you need to replace that filling. Every time you take a drill to a tooth, even if you try and be minimally invasive you will always make a bigger hole when you go back in. The best thing is not to start. (NZ6)*

### Goals

Approximately half of the NZ dentists made a comment that was grouped under one or more of the six constructs associated with goals, while fewer of the German and Michigan dentists made such statements. There was a marked overlap between the goals and intention domains, with an example of action planning being.

*… the decision ultimately is the patient’s and what they’d like to do. I merely just give them information and recommendations, and you know more often than not they defer to the practitioner anyway. Ah but I do feel you know an obligation to help these patients be as healthy as they can and in my opinion being minimally invasive, or being conservative, you know preserving their natural tooth as long as possible without negative outcomes is important. (USA7)*

### Environmental context and resources

Environmental context and resources comprised one of the larger domains for responses. Most comments were grouped under resources and material resources, followed by environmental stressors and organisational culture/climate. Comments categorised under resources/material resources generally centred not only on the lack of financial reimbursement for NI/MI but also on regulatory restrictions.

*There’s…a lot more advantages for the dentist to cut a filling ‘cos you, you get one…you get the fee for doing the filling and then two, eventually you get up to get the fee for replacing that filling at some time in the future. And then maybe 20 years down the track you end up having to do a larger restoration or a crown when the cusp cracks (NZ4)*

*It isn’t paid. It requires more work that is not paid. Hence it isn’t really worth it for me. Maybe for the patient but sadly not for me. It just isn’t paid well. (G4)*

Comments under organisational culture and climate pertained to colleagues supporting the use of NI/MI, or vice versa, with the organisational culture/climate being unsupportive.

*My hygienists are very preventive orientated you know and they’ve kind of encouraged the adoption of the fluoride varnish and… so I guess that was an enabler for me that my staff was gung ho. (USA5)*

Comments under enablers mentioned specific technologies (such as digital radiographs, magnification).

*Digital x-rays and being able to show people x-rays that they can see without having to look through a magnifying glasses and things, that makes it very real for people. So having sharp clear x-rays um, that are life size or bigger than life size that patients can see clearly and you can demonstrate progress and show them also. That, is the biggest educational tool ‘cos we’re living in a digital age where seeing is believing. (NZ4)*

Some Michigan participants also mentioned that instructing patients on NI was time-consuming, with patients reluctant to attend the required multiple appointments; in addition, NI/MI were good for patients with low pain thresholds or who were anxious.

### Behaviour regulation

Behaviour regulation was the domain with fewest responses, possibly because most participants were already using NI/MI. One example, however, of how participants were motivating others was as follows.

*So in our practice … we’ve set about just recently on actually rewarding each other within the practice using a card reward system where if you see another member of our team not doing standard brush and floss-type stuff but actually tailoring the message to that individual patient um with something that’s much more achievable. (USA13)*

### Barriers and enablers across different countries

Six barriers were identified. One of the most commonly mentioned included a lack of (or less) remuneration for NI/MI under different dental health funding schemes.

*National health insurance does not pay for non- or micro-invasive measures. There is no real financial support for it and…that’s why, speaking from an economical perspective, support (from the national health insurance) will only happen once we start using the drill. (G3)*

*Unfortunately the financial advantage is kind of negative because I'm financially advantaged to go in and do invasive… care, I’m not saying that that’s a good thing but when you look at it strictly like that. (USA7)*

Time pressure to perform NI/MI procedures was also mentioned as a common barrier, more likely to be mentioned by the German and US participants.

*The disadvantage is that minimal invasive treatments require more time, compared to those that are not minimal invasive. (G8)*

*So I guess sometimes the barrier can be time if a patient needs extra time and educating. (USA4)*

Not undertaking continuing professional development and basing treatment on their initial dental training was a further barrier, but identified only in the German and US participants’ responses.

*I really can’t tell you. I haven’t looked at any studies for 20 years. (G1)*

*The dental education when I was in Dental School. (USA11)*

A sense of anticipated regret (anxiety about not restoring an early proximal lesion before it progressed) was another barrier.

Dentists’ beliefs about whether patients were likely to adhere to oral hygiene instructions in the future and whether they were high-caries risk—as well as having unsupportive colleagues and/or practice leaders who were not interested in such methods—were two further barriers. Although most said their colleagues supported NI/MI management, there were exceptions. In some cases, these unsupportive colleagues were in a higher status position.

*So [name] is in his late sixties now. I think it would be fair to say and think he’d probably agree that he can’t be bothered with [preventive measures] anymore. (NZ5)*

A similar number of enablers were identified; one was the belief that remineralisation can occur in non-cavitated proximal lesions and that those can be arrested.

*It’s all about seeing evidence of arrested lesions, evidence of decalcification that has remineralised in people whether it’s radiographic or pictorial evidence, it might require both, but you’ve got a widely held and standard procedure dentistry for decades that a lesion that has not penetrated DEJ can remineralise and stay that way for 75 or 100 years if its properly cared for. (USA2)*

The understanding that restorations serve to weaken tooth structure was also a second identified enabler.

*Directly to avoid having restorative work done on their teeth and preserving the natural tooth structure because it’s widely accepted that once a restoration is placed in the tooth…it’s going to lead to a lifetime of more restorations, they’re going to get larger and larger and maintaining… existing tooth structure should be of paramount priority. (G2)*

Undertaking ongoing professional development and continuing education (and maintaining membership in professional groups) was another enabler.

*The dentists that I meet up with at advanced training/education courses and attend advanced training/education courses together or that I meet there are all, working [on] the same concept [incorporating NI/MI] as myself. However, we are not representing all the dentists. Sadly this is how it is. (G5)*

Having supportive practice colleagues and skilled dental team members who work collaboratively to undertake non-invasive procedures (such as cleaning and scaling) was another enabler. Some mentioned the significant role of dental hygienists, prophylaxis assistants or dental nurses in undertaking NI/MI methods, as well as alerting dentists and patients when they had non-cavitated lesions that needed monitoring.

*What we normally do is, oral hygiene instruction with flossing keeping the area clean, this is usually reinforced by the hygienists as well and also a fluoride application. (NZ6)*

*My hygienists are very preventive orientated you know and they’ve kind of encouraged the adoption of the fluoride varnish and… so I guess that was an enabler for me that my staff was gung ho. (USA5)*

Having the necessary finances, equipment and staff resources was also identified as an enabler.

*I think … digital x-rays and being able to show people x-rays that they can see without having to look through a magnifying glasses and things, that makes it very real for people. … and also just the immediacy of a digital x-ray, which pops up on the screen and having good, clear x-rays. Not sort of spotty, murky, fuzzy ones. So having sharp clear x-rays … that are life size or bigger than life size that patients can see clearly and you can demonstrate progress and show them also. That, is the biggest educational tool ‘cos we’re living in a digital age where seeing is believing. (NZ4)*

There were similarities and differences across countries (Table 3). The lack of remuneration or time was mentioned by nearly all; insurance systems (public or private) restrict remuneration for NI/MI to children or adolescents, with higher remuneration for restorations. The US dentists, notably, mentioned that placing restorations early on would enhance their survival chances, which is relevant given that dentists guarantee survival for some time (the same regulation is in place in Germany).

**Table 3:** Similarities and differences between dentists in the three participating countries. (−) Barriers, (+) enablers

Themes	New Zealand	Germany	USA
(−) Lack of or less remuneration for non/micro-invasive measures and healthcare regulation	Oh well actually one of the things that does come to mind is the way that the fee structure is set up for the adolescent children, the contract with the Health Board, I mean that is an external factor I suppose because if a person is looking to just for income and they see a number of small spots on the enamel you know they may decide, I mean that is a clinical judgement but the way the fee structure is set up there’s nothing for doing fluoride treatment but there is something for doing fillings. (NZ2). There’s… a lot more advantages for the dentist to cut a filling ‘cause… you get the fee for doing the filling and…eventually you … get the fee for replacing that filling at some time. (NZ4)	The flip side of the coin is the economic situation. National health insurance does not pay for non- or micro-invasive measures. There is no real financial support for it and hmmm, that’s why, speaking from an economical perspective, support (from the national health insurance) will only happen once we start using the drill. (G3) It’s really only the invasive measures that make it worth it. You get more money with it. It’s not like the national health insurance covers non-invasive treatments. Unbelievable. (G12) Non-invasive procedures are desirable for the patient and of course are very good, but in reality, one has to place some fillings (G3)	There’s also another thing that I’ve encountered is with a lot of insurers now, they have a delay before they would allow a new restoration to be placed in the tooth so I think that kind of pushes providers to take an incipient lesion and restore it. (US12) Well… unfortunately the financial advantage is kind of negative because I’m financially advantaged to go in and do invasive… care, I’m not saying that that’s a good thing but when you look at it strictly like that… (US7)
Mentioned (%)	83%	100%	70%
(−) Time to enact non/micro-invasive measures	I mean some procedures can be more time consuming, smaller and fiddler to do than cutting a nice big hole in the tooth. (NZ8)	G8: The disadvantage is that minimal invasive treatments require more time, compared to those that are not minimal invasive. (G6)	So I guess sometimes the barrier can be time if a patient needs extra time and educating (US4).
Mentioned (%)	8%	42%	33%
(−) Anticipated regret	Sometimes you will see a cavity and it is small and you think, oh I should drill that and then you second guess yourself and say, you shouldn’t. Then two years later they are back again and you think, oh thank goodness, I didn’t drill that and other times there is a massive cavity there and you bitterly regret your decision. (NZ1)	The disadvantage is that if one cannot see the patient for a follow up then it can turn to custard, rather quickly and 2 years down the track the next dentist would say: “This dentist had used micro-invasive treatments and now the tooth needs to be taken out, because no one removed the decay”. That is why I would be careful. (G1)	I might find that some of these things come back to bite me or patients who…really haven’t been able to change to oral hygiene… you change your eating habits or what not and now I see the patient back in two years and they didn’t come back in six months or what not and now we have got a tooth that needs a lot more work than if they were treated early on. (US18).
Mentioned (%)	92%	75%	23%
(−) Basing treatment on what they had learned in dental school and not undertaking ongoing professional development.	–	Int: Are you recommendations, the one you mentioned, based on studies? G1: I really can’t tell you. I haven’t looked at any studies for 20 years.	Int: Where did you get your knowledge for using non and micro invasive? US11: The dental education when I was in Dental School.
Mentioned (%)	0%	42%	23%
(+) Goal priority––non/micro-invasive measures	If I did do it I would be um, ah my patients would have more decay. I just think that enables success for my patients. I want it for my patients. I’m passionate about helping them to achieve oral, yeah that’s it really. (NZ3)	Well the biggest benefit is to the patient directly to avoid having restorative work done on their teeth and preserving the natural tooth structure because it’s widely accepted that once a restoration is placed in the tooth…it’s going to lead to a lifetime of more restorations, they’re going to get larger and larger and maintaining… existing tooth structure should be of paramount priority”. (G2)	US3: Yeah absolutely. I definitely like to be more on the conservative side. Int: Is it a priority in your professional role to do that? US3: Yeah, yeah definitely.
Mentioned (%)	83%	17%	15%
(+) Colleagues supportive of non/micro-invasive measures	Int: So your colleagues would be supportive of um non or micro invasive measures? NZ11: Incredibly supportive. In fact, we, we yeah, no that would be yeah, it would be looked down upon and discussed if there was a choice to fill and yeah certainly people would want to have a discussion about it and it would be an interesting point though, as I said, it’s quite protective of the person and the tooth and what you’re doing and how you’re doing it and yeah. If there was a big crack there and there was a big R1 and there was an occlusal and you know the, it might be that we chose to add that onto the filling so I suppose I might at that stage.	G7: One would have to rather ask, what would prevent me from using non or micro invasive measures within my practice? But to answer your question, there is nothing... There is the patient, then there is myself and one has to fully inform and instruct the patient and that is simple a time factor, but one has to take this time. Int: What about your colleagues? Do they have the same opinion? G7: No. They have a similar attitude.	Well our practice is kind of unique in the fact that we are part of a large, I won’t mention the net, but we’re part of a large insurance group, and they have used our facility as a cusp model for trying different preventative measures. For instance, over the past couple of years we have documented that as the preventative procedures increase in our patients, the restorative has decreased. And we can show that on a graph which is particularly interesting to um insurance providers and to patients, it benefits the patient too, but the insurance providers want to know if there’s a value to that, and how they can assign a value to it. (US20)
Mentioned (%)	67%	25%	41%
Undertaking professional development–belonging to professional groups/study groups/professional discussions with peers/attending lectures or conferences	Int: Yep. No, that’s cool. So do you there’s strong evidence for the recommendations for that CAMBRA technique and acronym that you just said, do you think that there’s strong evidence behind that? NZ3: Yeah. There is. Yep. Int: Thousands of cases that I have been following the process for about 15 years and yeah Professor Featherstone from America presented a couple of years ago in Auckland…It was Minimal Intervention Dentistry Conference.	Well. The dentists that I meet up with at advanced training/education courses and attend advanced training/education courses together or that I meet there are all, hmm, working not the same concept as myself. However, we are not representing all the dentists. Sadly this is how it is. (G5)	I mean it’s covered pretty well in Dental School, CE courses, manufacturers raps, introducing new product, journal articles, clinical studies, things like that. (US12)
Mentioned (%)	92%	25%	85%

Anticipated regret was also a general theme, manifested in the fear of not having managed lesions early by placing a restoration. The hope of changing patients’ risk profile seems limited, especially among the German and US dentists. For many, placing restorations is the “safer” option.

Relying on knowledge from dental school was more commonly found in Germany: nearly half of the German dentists mentioned that their treatment philosophy generally reflected what they learnt at university. By contrast, undertaking professional development or belonging to professional educational groups or having discussions with peers was more common with the NZ and US dentists, with only a few German dentists mentioning it. Having colleagues who supported NI/MI was another enabler, mainly among the NZ and US dentists.

Having the goal or priority of performing NI/MI was also a relevant enabler, mentioned by most NZ dentists, but only a few German and US dentists. Those who mentioned it all intimated that they wanted to do the right thing for their patients and felt good when it succeeded.

## Discussion

Two major groups of factors influenced dentists’ behaviour. The first was associated with knowledge, skills and the theoretical understanding of how to best manage dental caries and caries lesions. An awareness of the scientific literature—of the benefits of implementing NI/MI, and what these methods involve—was an enabler [7, 27]. Moreover, the experience that remineralisation can occur in non-cavitated lesions with fluoride application and dietary changes positively affects attitudes towards NI/MI [28, 29]. Having positive experiences and knowing about the research data could help to alleviate the fear that non-restored lesions could progress. This is relevant because many dentists (especially the US and German dentists) found restorations to be the “safer” treatment option, despite usually being aware of the long-term problems arising from restoration placement. This contradiction was even more strikingly for high-risk patients, where restorations have especially poor outcomes [16], while MI treatments have been especially successful here [7]. Another relevant aspect was the patient as a possible risk factor to be considered. Poor “compliance” and unmanageably high-caries risk were commonly cited reasons for not using NI/MI. Many dentists felt somewhat unable to reliably manage patients’ behaviour. This was more pertinent in Germany and the USA, while NZ dentists were generally more optimistic that they could do it.

The social aspects also shape behaviour, which may impact within the practice or the wider healthcare setting. For example, mandating guarantee times on restorations incentivised restorative overtreatment, shifting priorities towards restoration longevity, not patients’ health or tooth retention [21, 30, 31]. Another important barrier was NI/MI being remunerated only for children or adolescents [15, 32]. Such a restriction goes against long-term cohort data showing that the caries increment (number of surfaces newly affected/year) remains largely constant through life; caries is not just a disease of childhood or adolescence [33]. Moreover, all three dental healthcare systems appeared to pay better for restorative interventions than for NI/MI. Hence, dentists felt unable to provide NI/MI in some cases because this would not cover their expenses. Delegation of NI/MI procedures to auxiliary staff could improve NI/MI profitability in dental practice: the cost-effectiveness of NI/MI is very high in school or other non-practice settings [34]. Indeed, sharing responsibilities among the dental team has generally been found to enable less invasive caries management [15, 35].

We identified a number of aspects to be targeted by interventions to help close the implementation gap. First, dentists should be encouraged to regularly undertake continuous professional development (CPD), particularly in cariology [35]. Second, pre- and postgraduate education should focus more on dentists being not only just skilled treatment providers but also effective patient communicators [15, 35, 36]. If dentists could communicate effectively with patients about preventive techniques and behaviour change, the risk of anticipated regret and the associated wish to over-treat to be “safe” might decrease over time. Third, remuneration and regulatory practices should be aligned with current evidence. Outdated financial incentives favouring restorative or even prosthetic care will perpetuate outdated and (in some cases) harmful treatment approaches. Instead, remuneration and regulation should have appropriate incentives. Finally, there is a need for tools to easily, repeatedly and validly record and monitor early lesions. Since radiography has limitations and also requires high standardisation and the use of ionising radiation, technologies such as light transillumination may become more useful [37].

This study has a number of strengths and limitations. A large and diverse range of dentists from different countries was interviewed, allowing exploration of “drivers to practice” among countries and understanding setting-specific factors. The TDF provided the underpinning theoretical structure; while such a framework is advantageous [32], its use may have had unintended consequences (for example, findings are over-determined and/or alternative interpretations are left unconsidered) [32]. For instance, some comments were grouped into more than one domain, which may have resulted in more focus being placed on those than was warranted. To date, no studies have examined the fidelity of TDF application or those unintended consequences. Moreover, this was a qualitative study using interviews; future studies should aim to triangulate findings using other approaches. This would be in line with recent calls for triangulation of data using the TDF from questionnaires, interviews and observation [26].

## Conclusions

The decision on how to manage non-cavitated proximal lesions was influenced by numerous factors, and these differed to a certain extent among the three countries. As barriers to NI/MI, we identified lack of time or remuneration, basing treatment decisions on knowledge that is out of date, and a lack of patient “compliance” and involvement in decision-making. Enablers were undertaking ongoing professional development, being part of a professional group and working in a supportive environment. Financial reimbursement systems should fund NI/MI so that its clinical uptake is enhanced. Licencing bodies should insist on appropriate professional development in cariology or preventive dentistry as part of dentists’ ongoing registration. Undergraduate dental education programmes and CPD courses are the key to instilling shared decision-making principles as a cornerstone of the dental consultation.

## Additional file

Additional file 1: Interview guides and examples of comments grouped under the different domains and constructs. (DOCX 46 kb)
