# Supplementary material for: To fill or not to fill: a qualitative cross-country study on dentists’ decisions in managing non-cavitated proximal caries lesions
Source: Implement Sci. 2018 Apr 6;13:54. doi: 10.1186/s13012-018-0744-7 (PMC5889601; doi:10.1186/s13012-018-0744-7)
Supplement: Supplementary file 1 — Interview guides and examples of comments grouped under the different domains and constructs. (DOCX 46 kb) [file 13012_2018_744_MOESM1_ESM.docx]

# Appendix 1: Interview Guide

**Welcome and establishment of ground rules**

Participants will be thanked for agreeing to take part in the study and sparing time for the interview. They will be reassured there are no right or wrong answers. It will be explained that the use of a tape recorder by the researcher is to help them remember what is said without them having to take notes. Participants will be assured that the researcher will treat the information given as confidential.

The interviewer will then inform the participant about the focus of the study which is on **gaining more information about the barriers and enablers for dentists in using non- or micro-invasive measures for managing proximal lesions confined to the outer half of enamel and at the enamel-dentine junction. Non- or micro-invasive measures include applying remineralizing agents (fluoride varnish, CPP-ACP etc.), proximal sealing or caries infiltration, flossing and/or demonstrating oral hygiene maintenance.**

Thinking generally in relation to these recommendations, please explore the following. There are no right or wrong answers!

The interviewer will be open to the participants’ narratives and flexible in switching between the interview topics. The following is therefore a guide based on the domains from the revised theoretical domains framework. Although all domains need to be covered, the interview can be flexible in their approach to the interview structure.

**Background**: To begin, participants will be asked:

- Job title, years of experience since qualification
- Brief synopsis of place of work (solo/group practice; private - insurance/public mix; rural/urban; number of patients registered; number of dentists/hygienists in practice; remuneration system e.g. fee-for-service, capitation)

**Current Practice/Skills:**

- Ask the participant to describe the routine care they’d provide to manage proximal lesions confined to the (1) outer half of enamel and (2) at the enamel-dentine junction) in the permanent teeth of an adolescent or adult.
- Ask the participant whether there has been a situation where they have decided to restore a proximal lesion confined to the (1) outer half of enamel and (2) the enamel-dentine junction rather than carry out other, non- or micro-invasive measures as a first step?

Prompt: What were the circumstances? Why was the decision made? Is this situation common?

**1. Knowledge and Skills**

Are you aware of any guidelines in relation to non- or micro-invasive measures for the management of proximal lesions confined to the outer half of enamel (or at the enamel-dentine junction)?

- If yes, what is your understanding of the recommendation for management of permanent teeth?
- How strong do you think the evidence is for the recommendations? Is there anything that would give you more confidence in the guidance?
- Does the guidance help you give non- or micro-invasive management to patients? Why or why not?

2. **Intentions/ Social/Professional Role and Identity**

Do you view it as your responsibility to ensure non- or micro-invasive management is carried out in every situation possible? Is it a priority for you in your professional role?

Is management with non- or micro-invasive measures rather than restoration something that you intend to do wherever possible in the future? If yes, explore whether this maps to current practice. If no, explore why their intentions aren’t in line with guidelines.

**3. Goals/Behavioural Regulation**

Are non- or micro-invasive measures part of a routine you have for managing all patients with proximal lesions confined to the outer half of enamel (or at the enamel-dentine junction)?

Are there procedures or ways of working that would make it easier using non- or micro-invasive measures as a ‘first step’ rather than restoring proximal lesions confined to the outer half of enamel (or at the enamel-dentine junction) (prompts: training needs, courses; guidelines)

**4. Beliefs about Consequences/Reinforcement**

What are the benefits/advantages of using non- or micro-invasive measures as a ‘first step’ instead of restoring lesions at the (1) outer half of enamel and (2) enamel-dentine junction lesions? (prompt: To you? Your patients? Time? Staff resources? Financial incentives/disincentives? Prevent caries?

Are there any disadvantages/downsides of using non- or micro-invasive measures instead of restoring proximal lesions confined to the outer half of enamel (or at the enamel-dentine junction)?

Do you think the benefits of non- or micro-invasive measures outweigh the costs?

**5. Environmental Context and Resources**

To what extent do factors within your practice influence your ability to use non- or micro-invasive measures?

- physical resources (e.g. access to equipment; more staff/space)
- finances (e.g. time available; remuneration)
- colleague’s expectations, beliefs, attitudes etc.

(prompt: which factors act as barriers and which as facilitators?)

What factors related to your patients may influence your decision?

- co-operation, expectations, beliefs, attendance record, oral hygiene etc.

(prompt: which factors act as barriers and which as facilitators?)

What about factors outside your practice influence your decision whether you use non- or micro-invasive measures? (e.g. dental association, health policy, performance targets)

(prompt: which factors act as barriers and which as facilitators?

**6.     Social Influences**

Is non- or micro-invasive management something that your patients want?

Is managing proximal lesions confined to the (1) outer half of enamel and (2) enamel-dentine junction by non- or micro-invasive actively supported by colleagues in your practice?

-       In what way does the wider dental profession influence your decision about preventive management?

**Other**

Is there anything else about the non- or micro-invasive management of caries that you would like to mention that we haven’t already covered?

Is there anything that you have found the most helpful in assisting you in adopting non- or micro-invasive caries management?  (if haven’t adopted – what would be the most helpful in assisting you?)

Who would you trust/consider to be an expert/leader in the non- or micro-invasive management of caries?

**Closing**

Participants will be asked if they would like to add any further information and thanked for the discussion. Participants will be de-briefed on the next steps of the research process. To include giving participants product voucher and recommendations on non-invasive management of lesions.

## Appendix 2: Examples of comments grouped under the different domains and constructs

| **Domain** | **Constructs** | | **New Zealand (NZ)** | | **Germany (G)** | | **United States (US)** | |
| --- | --- | --- | --- | --- | --- | --- | --- | --- |
| **Knowledge** | | Knowledge | | It is a big part of my practice and it is a big part of my promotion strategy because I have seen some stunning results with it, and also the electron microscope, electron micrographs are quite convincing. Independent research is actually really good. It has been part of my practice for a number of years now actually. (NZ1) | | The function of the fluoride has been proven. (G5)  I | | So we have a fairly robust caries risk assessment tool. If we have risk factors that are not balanced by protective factors, so we have more risk factors than protective factors we process them at moderate risk, and if they have no disease indicators and appear very healthy and a disease is either not present or has significantly decreased with the remineralisation for its part of the healthy family we call them low risk. (US13) |
|  | Procedural knowledge | | Well the thing is the proximal lesions are usually at the contact point between the two teeth, where the two teeth touch and get the material into that lesion, you have to sort of put the material between the teeth and then try and floss it into place and similarly with tooth mousse. (NZ2) | | Then I would draw his/her attention to dental floss and the interdental brushes. Then there is of course the diagnostic analysis based on the X-rays and from all this I come up with a diagnostic analysis and, sadly I, as a practitioner, have to say that only very few are able to optimize their (dental) situation to largely avoid the use of a drill... However, then the requirement also is to regularly have the teeth professionally cleaned and have regular checkups. If I then notice that it is working I lengthen the appointment intervals to a year, under the condition that the patient has his teeth professionally cleaned twice a year. | | So if the lesions are incipient, still totally in enamel my recommendations would be firstly to improve oral hygiene, so I’d offer oral hygiene coaching, brushing and flossing technique…actually wrapping the floss around the tooth inner-proximally to remove any bacteria, food debris that is trapped in there around the tooth. Ah the second thing we would do would actually be to review the decay process. So how tooth decay works, specifically, consumption carbohydrates and in regards to frequency and duration, so what they’re eating and drinking, how they’re eating and drinking it, and specifically to avoid sipping and nibbling and extending those consumption times. And then we would make some recommendations for… fluoride toothpaste, a fluoride rinse, we may recommend fluoride varnish. | |
|  | Knowledge of task environment | | I would then talk about their diet, especially referring to drinks. Um with regard to the fluoride toothpaste, if there was ah, if there were a whole lot of them around the mouth and um, I would look at perhaps upping the fluoride concentration. And then if they’re going to be seeing hygienist, then I would get them to do one as well. (NZ5) | | I am a bit hesitant with elderly patients, [but] with younger ones I tend to check their general oral hygiene first and then I would check the papillary bleeding, well the papillary bleeding index. And if they have heaps of plaque everywhere, then I would check whether or not I will ask the patient to have a prophylaxis appointment. And I would decide afterwards and again will ask for them to come more frequently to see whether or not the situation has improved and then I would be more conservative and wouldn’t drill… (G4) | | The factors that will influence it is the patient history. Does this patient have a lot of dental work in his or her mouth or not? Obviously if someone comes in and they’ve already had 20 fillings done I’m going to be a lot more liberal in what I interpret radiographically as a problem than if the patient comes in with no dental work in their mouth. A perfect set of teeth and I see a little incipient lesion on a radiograph I’m not going to do it. I’m going to wait and see what it looks like the next time I take radiographs. (US19) | |
| **Skills** | Skills | | The comprehensive exam I found has worked really well. It’s a one-hour discussion and…I ask them a series of questions about what they’re health, what problems are they having. What treatment have you had in the past, have you had trouble at the dentist before, how do you look after yourself, let’s look at your medical history, let’s test your saliva, what do you drink when you’re thirsty, …how, did I say how do you look after your teeth…, what are your expectations of your teeth now, how long do you hope to keep your teeth? (NZ3) | | [In the] Outer half of the enamel, oral hygiene instruction?... I would not touch it and I would make sure the patient, I would…do a saliva test and quite a comprehensive exam initially. And so that would be an assessment of their, I ask questions about their diet and their cleaning habits. So fluoride toothpaste, flossing, interproximal cleaning…and then with their diet and I’d make, I’d be heavy on…just keeping the acids to meal times only and then, and allowing a period to neutralise. And, then … it depends on whether they have other, whether they have other serious lesions. Like if they have…deeper decay in other teeth then I, if they do then I would be, I would put, if I thought so, I wouldn’t definitely put them on tooth mousse. And if they had other decay, like bits in the dentine, then I would be doing, I do the chlorhexidine seven days a month fluoride 5000 twice a day. (G3) | | There’s no hard and fast rule. If somebody’s looking at it and when you do research I don’t think you’re going to come with a check list here because it just really depends on your relationship with the patient, trying to figure them out, trying to figure out compliance, trying to figure out their dietary habits and all that and then putting it all together, then you feed all that into the computer and you come up with your own answer of how can I best treat that patient. (US19) | |
|  | Skill development | | I mean you don’t come out of Dental School having done lots…of things. You have done a few of many things, and so you, the real learning starts, I mean certainly from personal experience in that first year out, where you become exposed to techniques you know, modern and preventive techniques that you might not have even heard of. I came out of Dental School and really it was brush teeth, floss your teeth and we will paint fluoride stuff on, but we have come a long way since then. (NZ8) | | Well I have been working as a dentist for ages and am increasingly treating my patient using that concept. Well not in the beginning, seen as I had learnt about this in a very different way, however, due to the regular courses I attend and due to my personal experience, I have learnt [a lot] and observe my patients and it was worth it. If one uses fluoridation treatment and if one has a good compliance and regularly attends prophylaxis appointments, then one can prevent micro-lesions from demineralization any further. (G5) | | I approach this, and I can’t speak for everybody, but I have a feeling that most practising dentists which I was for 35 years would first make the decision as to whether the radiographed proximal lesion was close enough to the dentine enamel junction to warrant being restored physically, to drilling it out and filling it. After that decision is made radiographically in one appointment, after that…if the decision is not to invade then yes, I automatically used to consider what else I could do to try to prevent the lesion from getting worse. Whether I think it’s close enough to the dentine that it’s already gone into the dentine clinically and it just hasn’t completely shown up yet radiographically…The decision is based on my judgement, my experience, and I’ve looked at tens of thousands of x-rays in my career, it’s based on do I believe from my experience. (US12) | |
|  | Competency | | Int: To what extent do factors within your practice influence your ability to use non-or micro-invasive measures?  NZ10: Probably just the sort of the, my previous experience in sort of rates of success and what not and sort of going down that track that I guess over time you sort of gain a feeling for which lesions you think will sort of respond to a non-invasive from a treatment and which ones you think are going to need more invasive treatment. | | Int: Would you change your treatment depending on adult teeth or those of youth? Are there differences?  G3: Yes, there are definitely differences. Regarding the youth, for example, it’s very possible to educate them. One would probably be able to manage the oral hygiene habits. However, if the patient is 70 years of age, then if you tell him to use floss, after 50 years of never doing so then he would most likely not do it. One would also have to think about the technical aspect, for youth the pulp is bigger, meaning it can become hypersensi[tive] if one drills too extensively. | | Int: How often do you use the fluoride products and which ones do you use?  US16: Just about all the kids get fluoride varnish and every six months when they come in unless they’re low caries risk, and I would say that many of my adults that are having problems with active decay or dry mouth or have had a lot of crown and bridge done will be using a prescription fluoride, either Clinpro 5000, one of the Prevident products if they have dry mouth they’ll use one of the Prevident fluorides that also has potassium nitrate…. So you know we do use Clinpro, Prevident frequently and the varnishes and sometimes silver diamine fluoride too. (US16) | |
|  | Ability | | The routine care at the enamel dentine junction, once again I alert them and then I just make an educated guess to be perfectly honest about the actual…I always look back to previous x-rays to look at the rate of progress or change. And that determines where I go from there. If there’s been no change, I will have it on a monitoring basis and the card gets marked with a ‘watch’. And then I will reiterate the importance of flossing and it’s, and often, and sometimes I’ll tell people if they’re flossing infrequently that they can make that flossing more effective by usually putting a smear of toothpaste on their finger, smearing it along the teeth and flossing that between the teeth, to, provide some sort of abrasive, to help remove hardened plaque and…leave behind some fluoride that isn’t already contained in the paste. (NZ4) | | Um, some people talk about fluoride um, application of fluoride between the teeth. Um, just having an um, there is also the anti-microbial approach using chlorhexidine to reduce the amount of bacteria between the teeth. So, um, I use that technique to um, it is more targeting the periodontal problems but ah it does have the side effect of also targeting um, reducing caries between the teeth as well. Then there is the high fluoride toothpaste that we provide some people with a very high caries rate to sort of turn the tide for them a bit, particularly when we know that hygiene is never going to be a great feature. (G10) | | But again it’s really a gut feeling I can take… you could show me the exact same x-ray and in one patient I may be drilling on that tooth and in another patient I may not be drilling on it. So I don’t have the check list that I can say a lot of it is your experience you’ve built up over a period of time and sometimes I’m right and sometimes I could be wrong. So but I follow my best judgement. But it’s not a written list in essence. It is the 36 years of experience I have in dealing with the management of caries. (US18) | |
|  | Interpersonal skills | | So when you can show what’s going on, and then explain how it’s going on, they can almost work it out for themselves. And I find most people do…I had a lot of success just not underestimating people’s intelligence. You know finding out what they know, if they’ve done a bit science at school and we talk about pH with school children. (NZ4) | | When we treat children we don’t ask for an additional payment. On top of that we have very sophisticated patients, that value this and have trust, because we don’t always use  invasive measures straight away.  (G11) | | I think my staff, given a whole hour, while I’m waiting for anaesthesia or given a whole hour for hygiene there’s plenty of time to talk about what to do. So tell me about your day, tell me about your habits. No, we usually ask them what their regime is like. I mean there’s plenty of time. We know we don’t make money, yeah, and we’re not a rush, rush, rush clinic. It’s not get ‘em in, get ‘em out out of it, but people like to feel like you’re educated and up on the latest research. (US5) | |
|  | Practice | | Well the, the routine care at the enamel dentine junction. Once again I alert them and then I just make an educated guess to be perfectly honest about the actual; I look, I always look back to previous x-rays to look at the rate of progress or change. And that determines where I go from there. If there’s been no change, I will have it on a monitoring basis and the card gets marked with a ‘watch’. (NZ4) | | If I, have the feeling that fluoridation is enough then yes. However, I have to have the feeling that something is changing in the patient’s life. So the decay has appeared, then spread and if I then notice, from experience, that the (patient) has grown up and has improved oral hygiene, (and) his nutrition, then I would continue to take less invasive measures. (G1) | | You know I don’t know that there are guidelines out there. I think it’s more of a clinical judgement of what you’re seeing and what you’re looking at. If it’s not through the DEJ I think that’s a call you have to make in talking with the patient. (US15) | |
| **Social influences** | Social pressure | | At the enamel dentine junction, it becomes more… How compliant are they going to be with the products? If they’re not interested in the products at all and they have terrible oral hygiene then …I’d be more inclined to, if things start to head into the dentine to expect them to, expect the cavities to flourish, to grow fast rapidly. So I would need to take other, we would need to do other things… it’s just not going to work. (NZ3) | | I think (the treatment) would be depending on the overall oral hygiene of the patient. If the patient has great oral hygiene, apart from, for example, 1-2 interproximal lesions that extend up to the inner enamel part, then…if he has otherwise a great oral hygiene, then I would not consider invasive treatments. (G2) | | Yeah most, almost all of our patients are very accepting and appreciative of our… more conservative or non-invasive techniques to try and limit their caries, and so I don’t think that cooperation or their effort is limited at all. I mean they’re on board 100%. (US7) | |
|  | Social norms | | NZ6: I suppose other colleagues that are not within the practice but… ex-class mates or ex-University mates who are dentists as well, that I would catch up from time to time at social gatherings.  Int: People who are like minded?  NZ6: Yeah, people who are like minded because…we tend to just congregate together and um, and talk about the same things and we generally also, because we think the same, we practice the same way. | | Int: What about your colleagues? What do they think?  G4: Well. I do think there are colleagues that rather do invasive treatments and place more fillings than necessary and then there are those colleagues that always observe and are not getting anywhere. Yes. Well I do know, the ones that are close to me think the same way I do. | | Well from my experience… talking with colleagues, I think there is… I think most of the colleagues I hang out with and talk with would rather never have [drilled]. I mean because you know we all feel that once you cut that tooth you’ve changed the makeup of that tooth and you’ve weakened that tooth, and you really haven’t done that tooth any favours by doing that except arresting the decay which was going to go rampant. So I would say that… most people like to be… conservative (US15). | |
|  | Group conformity | | I thought well this is common practice, this is standard, … practice dentistry now so I’m not going to do this treatment thinking that I’m being out there and a fringe dentist. (NZ3) | | Int: What about your colleagues? What is their opinion in regards to this?”  G5: They use the same concept, there is consensus. | | It is the responsibility of the profession in general to to [use] the least invasive option when it’s more appropriate. (US1) | |
|  | Social comparisons | | Education and peer support, I mean I tend to move in circles, in like-minded circles. I am a little intolerant of people practising in the dark ages, I mean archaic dentistry is still appropriate in some situations, the smaller holes the fewer holes. | | Well most colleagues think that invasive measures generate more income than non-invasive measures, that’s why most [colleagues] wouldn't consider non-invasive treatments, because I have enough patients and enough work I tend to do drilling as little as possible (G2). | | I happen to use rubber dam isolation as much as I can but on almost, even on a crown preparation, I bond my core in and do most of my preparation with a rubber dam on, and I’ve done that ever since I got out of school but I don’t think that a lot of my colleagues, I don’t know what the report is, it would be an interesting statistic to know but I don’t, I’m not under the impression that the majority of my colleagues use rubber dam isolation which I would think would make micro-invasive proximal lesion treatment pretty difficult. (US9). | |
|  | Group norms | | It is a particularly interesting area, because you know 40 years ago, professional role we, if we found a cavity like that, we would generally cut a reasonably sized cavity and fill it with amalgam. (NZ2) | | When I do see (my colleagues), then I do notice an amazing knowledge in that area. There are a lot (of colleagues) that are pretty careful. | | Int: Are non or micro-invasive measures supported by your colleagues?  US3: Yeah it’s not a popular topic. | |
|  | Social support | | I think attending and belonging to a group such as New Zealand Minimal Invasive Dentistry has been probably the strongest thing ‘cause you’re mixing with a lot of like-minded colleagues and they most definitely do influence you and, and inspire you. And it becomes just a natural thing to move into that, that kind of approach. (NZ12) | | The dentists that I meet up with at advanced training/education courses and attend advanced training/education courses together or that I meet there are all, working not the same concept as myself. However, we are not representing all the dentists. Sadly, this is how it is. (G5) | | I talk to many dentists at study clubs and CE courses. (US16). | |
|  | Power | | Ah well, a lot of times I don’t actually give people a lot of choice. I’ll tell them what the best. (NZ4) | | - | | - | |
|  | Intergroup conflict | | Oh, the arguments dentist have about what to drill and what to drill. (NZ1) | | A friend of mine, who is also a dentist, had detected several typical wedge shaped defects interproximally on a bitewing X-ray of her patient. They extended into the enamel-dentine-junction or shortly before it. She recommended to the patient to treat those lesions minimal invasively. The patient got a second opinion… that dentist told the patient that there is no decay. So the patient took the matter to the dental association. (G8) | | I think the problem is bigger in that we have a bunch of dentists who are apathetic and truly only concerned about … I’m going to say it’s, about finances… and there’s no way to pay back student loans if people don’t pay you to treat disease… but you can pay back your student loans when you do more crowns. So getting dentists to really see themselves as a healthcare professional, in this part of a health care system, not just as a dentist, and if they could see that they are part of an entire team whose mission is to make people healthy, not just to do procedures I think that would be huge. (US13) | |
|  | Alienation | | It is pretty corrupt out there…they [the Government] said oh we are going to pay for, four occlusal restorations in 2015, I didn’t believe them because they never tell the truth. (NZ1) | | Int: So what you are trying to tell me is that you earn less money using non- or micro-invasive measures?  G7: Exactly. It’s always about money. When the patient goes to a different practice, because this one is closed, then the colleague says: “Oh what is this, look, there is more decay and what not.” And that is wrong and terrible. One would have to be able to understand that dentistry isn’t all about a burr and filling materials, but also how you would act in respect to the patient. And one does not drill a hole into everything. However, this understanding is still lacking. | | And that dumbing down in the profession bothers me in medicine, and it bothers me in dentistry and … we used to have a middle… we’ve always had a high end and a low end in dentistry but we had a real strong kind of middle class in dentistry, of a very good thoughtful dentist and they’re getting beat down by the lower reimbursement rates and the pressure from insurance that they’re having to accept more and more patients every day and they really don’t… they really stopped caring and not doing what they’re capable of doing. (US16). | |
|  | Group identity | | It is a particularly interesting area, because you know 40 years ago, professional role we, if we found a cavity like that, we would generally cut a reasonably sized cavity and fill it with amalgam. (NZ2) | | Well usually it is colleagues that have studied actually see this topic the same way. However, again and again I see colleagues that have got themselves financially into a difficult situation and seal everything that is possible and change fillings that don't even need to be changed. Black sheep are here and there and everywhere. | | I think in talking with colleagues and see what they are doing, see if there is any new techniques that I may not be aware of and the influence the decision making process but then once you have that information and then going back to the research (US1). | |
|  | Modelling | | Int: Is managing proximal lesions confined to the outer half of the enamel by non or micro-invasive techniques actively supported by colleagues in your practice?  NZ4: Well, actively supported? I guess so but I don’t really, I’m sort of, I don’t, I don’t rely, I don’t need any support to know it’s worthwhile. | | - | | The people that I hang around with and study with, you know we talk about this all the time, … And so… we think similarly, we struggle with…preventive all the time and we talk about it in our study club, and in other groups that I’m involved with…my peer group talks about it, but those are peers that have practices similar to mine… and so we tend to be fee for service outside of insurance networks. We tend to be … looked at pretty highly amongst the whole profession, and so you know that’s the group that I talk with. I do lecture from time to time and I sponsor lectures, so I see a real … cross section of other dentists, but I don’t know what their understanding is. (US16) | |
|  | **Professional identity** | | This is standard, this is standard practice dentistry now so I’m not going to do this treatment thinking that I’m being out there and a fringe dentist. Because if the Dental Association is printing it in their magazine, it’s standard practice so I can, I’m happy with that now. (NZ3) | | - | | I think you know, I have patients in all of those categories, three months, six months and then you have those that disappear for several years and return. I just think the time spent reviewing the treatment needs of the patient is beneficial, I’ve always seen it as part of our ADA Code of Ethics to inform and provide the patients with education and the opportunity to ask questions and then have, play a role in their treatment but doesn’t mean we’re always gonna get them to comply. (US12) | |
| **Social/ professional role and identity** | Professional role | | Well the routine care at the enamel dentine junction, once again I alert them and then I just make an educated guess to be perfectly honest… I always look back to previous x-rays to look at the rate of progress or change and that determines where I go from there. If there’s been no change, I will have it on a monitoring basis and the card gets marked with a ‘watch’. I will reiterate the importance of flossing. (NZ4). | | If it is decay in its early stage, that I can see on the x-ray. If [the decay] is within the enamel, then I wouldn’t open it… In those cases, I will check if the patient regularly has prophylaxis appointments and if they are willing to participate. If that isn’t the case, I try to remind them how important it is. (G5) | | It’s our job to inform. So, and make a recommendation. Everything is risk management. If you don’t do this you run the risk of it getting deeper, so high risk, low risk, minor risk. If you have three lesions you’re a high risk. But yeah it’s my job to educate. (US5) | |
|  | Social identity | | Int: Is managing proximal lesions confined to the outer half of the enamel and at the enamel junction by non-or micro-invasive techniques, supported by your dental colleagues?  NZ1: It probably is; we don’t talk about it a lot to be honest. I don’t know why we don’t talk about that. Nobody has raised the subject we talk about all sort of other things, interesting cases, all cases […] or implantology we talk a lot about that, yeah, all the new leading in picks but yeah, proximal lesions, no we don’t really talk about that. | | - | | - | |
|  | Identity | | - | | - | | - | |
|  | Professional boundaries | | I mean I tend to move in circles, in like-minded circles, I am a little intolerant of people practicing in the dark ages. (NZ8) | | Colleagues that have studied actually see this topic the same way. However, again and again I see colleagues that have got themselves financially into a difficult situation and seal everything that is possible and change fillings that don't even need to be changed.... (G2) | | Better financially would definitely be letting things grow because the bigger the problem the bigger the pay check. But we never, I was taught both in school and from my boss to never let money dictate how you treat patients. You should always do what’s best for the patient, which is how I would want my dentist to treat me if I didn’t know any better. (US17) | |
|  | Professional confidence | | Ah well, a lot of times I don’t actually give people a lot of choice. I’ll tell them what the best [treatment]…I wouldn’t, if it’s really something they need to do, I’ll do it… I’ve got a… pretty good bunch of patients there actually who trust my opinions. (NZ3) | | - | | I’m sure that it’s something because it’s one less procedure that they have to have, you know I think that…given an option, most people would choose not to have something done on their teeth if they don’t need it…I think that they rely on me or… patients rely on their own practitioner to say this is what you need and presumably they trust what you’re saying. (US10) | |
|  | Group identity | | If it goes beyond the enamel dentine junction you may at that point decide to … drilling it out and filling it, is an option you have got. It doesn’t mean you have to do it, or you should do it, it just means that you possibly should do it. It is a bit vague but caries is vague… Oh, the arguments dentist have about what to drill and what to drill. (NZ1) | | Int: What about the general dentist  community, what do you think is  their general approach?  G10: It varies. There are certain colleagues that go by how much they get paid off it, but would probably also do worse fillings. I am sure it is mixed. | | I think the opinions are right across the board…I think I could take 10 dentists, pluck them out of a general population of dentists, I might hear 10 different ideas, so I think I can find people who share my opinion and other people…because people come into my office, even in our own practice we’ve had student externs from the dental school in our city in their senior year and they will be doing restorations. And their ideas can be very different to my sometimes. | |
|  | Leadership | | NZ12: I have just said farewell to a colleague who worked with me for just one year and she was cutting everything that had even had a little micro dot a third of the way through the enamel. And I had to have a discussion with her, this is a young practitioner… [names university] trained, who had been…I think four to five years who was cutting everything. And, I had to have a word with her. In the interests of the patient I wanted to see patients of my practice treated as I would wish to be treated myself. | | Well I’d like to think that my colleagues respect my authority as a boss. But in principle we are on the same page. My colleagues think the same way. (G2) | | Int: And so when they have all those choice and they’re flooded with all that information, how do you, what do you think influences their decisions the most, who do you think or what sort of drives their choices when they’re flooded with all those choices?  US12: I think it's just the time spent by the dental hygienist and the dentist informing them and helping them go through what’s available to them to make the best choices. | |
| **Beliefs about consequences** | Beliefs | | I encourage people to use tooth mousse from GC this is the calcium floss faith, donator. And, just start with that really, I would never drill them. I believe good hygiene can often heal them on its own (NZ1). | | Int: Those were advantages for the patient. Are there are any for you?  G8: Yes. I can look into the mirror every day.  Int: Do you think the advantages outweigh the costs that are associated with this treatment?  G8: Yes, but it’s not too good for my wallet though. | | Conservative non-evasive treatment is the appropriate thing to do. (US3) | |
|  | Outcome expectancies | | Probably half of my day is restoring fracture fillings, fractured cusps, teeth that have blown up, extractions, front fillings, that sort of thing. It is sort of a relatively smallish part of my practice, sort of that really early sort of reversible sort of caries management but nevertheless ,I think it is a really important one … if there was sort of more minimally invasive treatment applied then maybe all of the subsequent issues down the track may not be necessary to the same degree. (NZ11). | | I absolutely can’t stand it if someone drills into one occlusal surface after the one, bam, bam bam. Yes. Hmm. My dad is also a dentist. He doesn’t say many smart things in regards to conservative dentistry, however, he is always right with one thing, wherever you had one filling, you will get a bigger filling and at some point a root canal. (G8) | | I think if you look back, I mean I’ve been 41 years, so back when I was at dental school if we saw a little etching of the enamel we restored it. You know the old philosophy. But … that breaks down to be a larger restoration, if that breaks down… so it sets up a domino effect to the point where you know it could start off as a small lesion at age 20, and by the time they’re 45 it could need a root canal or extraction. I mean if you can postpone some of that to a later age then the domino effect has a better… and if you look at the lifespan of the patient it might be a better choice to have that tooth at age 80 might be in better shape if we did the minimal philosophy at age 20. (US20) | |
|  | Characteristics of outcome expectancies | | Well, the biggest advantage (of non or micro-invasive measures) is that you may remove the need completely for an MO or a DO, and MO’s and DO’s are you know a major cause of loss of tooth strength, which when you treat patients all their lives you, it’s tooth fractures come back to haunt everybody eventually. And fractures are nearly always initiated by a filling. | | Seen as the tooth can be preserved as it is. In this respect it outweighs the cost. One would have to explain to the patient, that he would benefit more from it. (G9) | | Int: What do you think are the benefits of a non and micro-invasive measures?  US12: Preservation of tooth structure, elimination of the need to repeat a restoration a couple of years later, ...maintaining an easily smooth tooth surface for oral hygiene, improve oral hygiene by maintaining the natural tooth structure…restoration is never gonna be as smooth as the original tooth. | |
| **Reinforcement** | Anticipated regret | | I have some real doubts about the effectiveness of arresting a lesion at more of less at the dentine junction. I guess I have always been very disappointed that they come back in a year or so and you have got to go and do a slightly bigger filling than you otherwise would’ve done. (NZ2) | | Well the disadvantages are that one would risk the decay to go deeper,  the decay would advance further resulting in the inflammation of the pulp and in extreme cases the need for a root canal. (G3) | | I mean for the patient again, if it works well…they’re going to spend less money out of their pocket when they’re not doing more procedures, and the other benefit they get is teeth can remain more sound for the future. That being said, the disadvantage for the patient is that if the caries continues to grow…they might need root canal therapy at some point. (US19) | |
|  | Consequences | | Int: Are there any disadvantages or downsides of using non or micro-invasive measures instead of restoring proximal lesions confined to the outer half of the enamel?  NZ4: Ah well, potentially you may not see it for two years and that little, beginnings of that lesion could you know go to sort of, accelerate quite, much more rapidly than you think. So you might see them come back and there’s a thumping great hole where you just saw the beginnings of one that you might have underestimated its progress. | | Int: Is there something that strengthened your choice to use non- or micro-invasive treatments?”  G10: Yes, the success, that one has. It’s encouraging when you notice that there hasn’t been any change to the worse after  1, 2, 3 years. | | Nine times out of ten we say look demineralize this you can get your tooth to heal itself and we know that if we do that it’ll be stronger, we need to take x-rays in 6 months at least, if not sooner than that to determine that it’s just stopped, and if we see them progressing then we will let you know and then we’ll have to put a restoration in that tooth. But most of our patients come to our practice because they understand that and other than cutting the tooth first. (US13) | |
|  | Rewards | | So there and that is a big, it is a big part of my practice and it is a big part of my promotion strategy, because I have seen some stunning results with it. (NZ1) | | Int: What are the advantages if one is to use non - or micro-invasive treatment measures as the first part of the therapy instead of taking an invasive approach?  G1: Certainly for the patient, preserving tooth enamel. I tend to have a better feeling with it and sleep better at night. | | The benefit would be grateful patients, hopefully referrals. I’d say the only way for me to…have a successful long term practice is for you to stop flossing and brushing and start drinking Coke again, or just refer one of your best friends to us. (US17) | |
|  | Incentives | | I do see lesions disappear on x-rays, you know, with improved hygiene and those strategies, you see those little grey flecks that sit halfway through the enamel, you see… a year or two later or two years later is the average sort of interval between x-rays normally, you see them disappear. (NZ1) | | Respectively this would delay or eliminate the point at which one would really need the need to use invasive measures, which … ultimately results in the delay of losing that tooth. (G8). | | I mean because you know we all feel that once you cut that tooth you’ve changed the makeup of that tooth and you’ve weakened that tooth, and you really haven’t done that tooth any favours by doing that except arresting the decay which was going to go rampant. (US15) | |
|  | Punishment | | - | | - | | When we were taught once something radiographically hits the DEJ, that it actually is something that…can spread along the DEJ and should be restored ... If that is still the common feeling about that, if you don’t restore it and somebody goes, moves away and goes to a different dentist and says…you have these cavities here that should be restored, I mean are you remiss, have you not done your duty, I mean can you be accused of not practising to the standard of care. (US12) | |
|  | Consequences | | And also, practising good dentistry as a health service, ultimately the people that really matter will begin to notice and you end up having a full book for that reason. | | Non- or micro-invasive treatments require more time. But it is worth it for the dentist-patient relationship.  (G9) | | The biggest advantage would probably be preserving the natural tooth … there’s, many secondary benefits, one avoiding operative dentistry… getting anaesthetised and having to sit in the chair and then do that 5-7 years later and then do it 5-7 years later and then get your crown and then get your root canal in 10 years and then lose the tooth 20 years after that. (US7) | |
|  | Reinforcement | | Actually some of my private patients I do get to come back sooner and have them fluoride… And it’s quite exciting to think that it’s all stabilised which is all good. (NZ5) | | Preserving tooth enamel. I tend to have a better feeling with it and sleep better at night. Also I can complete the work faster. (G1) | | I mean to be honest it would probably be a financial disadvantage because you’d be doing less operative dentistry. But that’s not really my goal, my goal is actually to help people. I think and the staff feels that, so that would be the main benefit there in terms of being fulfilled in regards to our careers. (US7) | |
|  | Contingencies | | - | | - | | In trying to kind of manage a diet, trying to stress the importance of floss… (US8) | |
|  | Sanctions | | - | | - | | I think in some circles… once something radiographically hits the DEJ, …. it should be restored … if you don’t restore it and somebody goes… to a different dentist and says how come…you have these cavities here that should be restored, I mean are you remiss, have you not done your duty. (US12) | |
| **Goals** | Goals | | No, well, yeah I, I, I do it already. I really try to, not, I try to not intervene as often as possible. (NZ4) | | I have all the materials here. However, it is also a little bit depending on the decision time of the patient. If (s)he randomly turns up, then (s)he would first have to make an appointment or get something for pain relief. In the end it is also good for the dentist-patient-relationship when the patient knows that one is doing everything they can to preserve their teeth with the least invasive approach. (G9) | | I do feel you know an obligation to help these patients be as healthy as they can and in my opinion being minimally invasive, or being conservative, you know preserving their natural tooth as long as possible without negative outcomes is important. (US7) | |
|  | Goal priority | | I’m a rural practitioner and…I practice three days a week. So I, if I did do it I would be um, ah my patients would have more decay. I just think that enables success for my patients. I want it for my patients. I’m passionate about helping them to achieve oral [health], yeah that’s it really. (NZ3) | | Well the biggest benefit is to the patient directly to avoid having restorative work done on their teeth and preserving the natural tooth structure. (G2) | | US3: Yeah absolutely. I definitely like to be more on the conservative side.  Int: Is it a priority in your professional role to do that?  US3: Yeah, yeah definitely. | |
|  | Goal/target setting | | - | | - | | We’ve seen excellent results with my patients over the years with the (micro-invasive) techniques. And you know as new technology and techniques come out, new materials come out we keep looking for ways to help our patients to be more effective in that way. | |
|  | Goals (autonomous/  controlled) | | I think if I can communicate well enough, what’s going on, then they shouldn’t be feeling like that. Most people would prefer not to. I have had people though say just fill it and been really shocked and saying I can’t, I just can’t do it. (NZ6) | | - | | Knowing how short-lived restorations really are, I tend to be pretty conservative in terms of recommending them, unless you know they’re absolutely necessary I’d like to give the patient the knowledge and the tools to be able to save themselves from that downward spiral of filling after filling after filling. I guess my practice is geared more conservatively, preventively orientated to coach patients to avoid dentistry if we can help it. (US4) | |
|  | Action planning | | I would never drill them (NZ1) | | - | | Int: Do you think a conservative approach is your responsibility to seek out and ensure non or micro-invasive management in every situation possible?  US7: I would give… the decision ultimately is the patient’s and what they’d like to do. I merely just give them information and recommendations…but I do feel you know an obligation to help these patients be as healthy as they can. | |
|  | Implementation intention | | You should still monitor it and do the preventive steps. I really try and sit on them. I do feel a bit nervous about it with some of our patients because I have no idea if they’re going to come back and it worries me that they’ll come back in five to ten years with a big hole, but yeah I’m definitely more reluctant to fill. (NZ5) | | Int: To what extent does the current scientific knowledge influence your treatment in regard to interproximal lesions?  G11: Very strongly. The development is  going towards the preservation of teeth  and structures. | | So in our practice we want to make sure that you’re a healthy person, not just a healthy mouth and we give them the opportunity to set goals for themselves and then we reward them when they achieve those goals. US13) | |
| **Environmental context and resources** | Environmental stressors | | Oh well actually one of the things that does come to mind is the way that the fee structure. I mean that is an external factor… there’s nothing for doing fluoride treatment but there is something for doing a filling. (NZ2) | | In principle [non or micro-invasive measures] is a great idea, but it is not paid for by the national health insurance. The patients would have to pay it themselves and I would only do it for lesions in their beginning stage. If it is extending into the enamel-dentine junction, then I would not use Icon. (G3) | | And the insurance companies won’t pay for it. And if you’re always used to paying out, I don’t know …You’ve got to pay twice because I’ve got to make a living. Even if I’m honest and not greedy or anything. You’ve got to make a living right. (US1) | |
|  | Resources/material resources | | There’s…a lot more advantages for the dentist to cut a filling ‘cos you, you get one…you get the fee for doing the filling and then two, eventually you get up to get the fee for replacing that filling at some time in the future. And then maybe 20 years down the track you end up having to do a larger restoration or a crown when the cusp cracks. (NZ4) | | Time is always a factor to be considered. …if you have 30 patients, then you can’t do a filling for all 30 patients. Then you would use fluoride lacquer and tell the patient to come back... Our boss wants to make money (laughs). Non-invasive procedures are desirable for the patient and of course are very good, but in reality, one has to place some fillings (G3) | | Well you know, unfortunately the financial advantage is kind of a negative because I'm financially advantaged to go in and do invasive… care, I’m not saying that that’s a good thing but when you look at it strictly like that… (US7) | |
|  | Organisational culture/ climate | | Like most of them don’t even place fissure sealants in and that is really easy so, I don’t know, they are not into it, I don’t know. | | Int: To what extent do you influence certain factors within your practice  to use or not to use non- or micro-invasive treatment  options?  G10: Well firstly of course, that I am under no pressure or obligation from my employer to practice a certain way. Yes. And that I have access to all the resources and I can pretty much use the treatment approach/concept I want. | | My hygienists are very preventive orientated you know and they’ve kind of encouraged the adoption of the fluoride varnish and… so I guess that was an enabler for me that my staff was gung ho. (US5) | |
|  | Salient events/ critical incidents | | Oh, I think the downside will be, if the patient is not good with home care, not motivated to do what they need to do then these lesions can progress. …So, now for me if I feel the motivation to clean their teeth is not there, I think it is better for us to put a small filling in (NZ6) | | One cannot use preventive measures  without prophylaxis assistants. (G11) | | US6: I have had some conversations where colleagues have said that they are surprised that we do adult fluoride treatments. And I don’t know that that’s routine.  Int: What do you think they’re surprised about?  US6: … that I can get patient compliance with adult fluoride… | |
|  | Person-environment interaction | | - | | - | | So the barriers, I think the biggest barrier is the dentists’ themselves… I suspect that … they’re not used to commenting about it, and I don’t think they really know whether they’re preventing or not, and I think they’re so busy and so on with the practice, there’s too many patients, too many people that don’t have the time or the interest to collect the data to see what success they have. (US16) | |
| Behaviour Regulation | Barriers and facilitators | | I think ah, yeah well, digital x-rays and being able to show people x-rays that they can see without having to look through a magnifying glasses and things, that makes it very real for people. Saying and also just the immediacy of a digital x-ray, which pops up on the screen and having good, clear x-rays. Not sort of spotty, murky, fuzzy ones. So having sharp clear x-rays um, that are life size or bigger than life size that patients can see clearly and you can demonstrate progress and show them also. That, is the biggest educational tool ‘cos we’re living in a digital age where seeing is believing (NZ4). | | Int: Has there ever been a situation, where you decided to treat an interproximal lesion immediately, instead of firstly using non - or micro-invasive measures?  G4: Yes. That has probably happened to  a patient with lots of decay and poor oral  hygiene. And maybe (who also had) a  pain sensibility. | | Of course, it’s time saving, it’s less time on the chair and less time off school if there’s, you know an older if the patient’s in high school or less time off work (US11). | |
|  | Self-monitoring | | Int: And I’m going to ask you that question again at the enamel dentine junction?  NZ5: You should still monitor it and do the preventive steps...I really try and sit on them. | | - | | So in our practice we want to make sure that you’re a healthy person, not just a healthy mouth. (US13) | |
|  | Breaking habit | | Int: And I’m going to ask you that question again at the enamel dentine junction?  NZ5: I think I did prior to Lyndie coming to [town] to talk to us …. | | - | | So in our practice we want to make sure that you’re a healthy person, not just a healthy mouth. (US13) | |
|  | Action planning | | I would never drill them. (NZ1) | | Int: But if your patient has perfect oral hygiene, is very compliant and is presenting you with a non-cavitated decayed lesion that extends up to the enamel-dentine junction, then you would you would open it too?  G4: I would definitely prefer a minimal invasive filling. However, in individual cases I may leave the decision up to the patient, yes. But I would also choose to do a minimal filling here but if the patient is willing to wait then I would also wait. | | Knowing how short-lived restorations really are, I tend to be pretty conservative … I guess my practice is  geared more conservatively, preventively to coach patients to avoid dentistry if we can help it. | |
